# Heart rate and startle responses in diving, captive harbour porpoises (*Phocoena phocoena*) exposed to transient noise and sonar

**DOI:** 10.1242/bio.058679

**Published:** 2021-06-16

**Authors:** Siri L. Elmegaard, Birgitte I. McDonald, Jonas Teilmann, Peter T. Madsen

**Affiliations:** 1 Zoophysiology, Department of Biology, Aarhus University, 8000 Aarhus, Denmark; 2 Marine Mammal Research, Department of Bioscience, Aarhus University, 4000 Roskilde, Denmark; 3 Moss Landing Marine Laboratories, San Jose State University, Moss Landing, CA 95039-9647, USA

**Keywords:** Exposure, Sonar, Acoustic startle reflex, Habituation

## Abstract

Anthropogenic noise can alter marine mammal behaviour and physiology, but little is known about cetacean cardiovascular responses to exposures, despite evidence that acoustic stressors, such as naval sonars, may lead to decompression sickness. Here, we measured heart rate and movements of two trained harbour porpoises during controlled exposure to 6–9 kHz sonar-like sweeps and 40 kHz peak-frequency noise pulses, designed to evoke acoustic startle responses. The porpoises initially responded to the sonar sweep with intensified bradycardia despite unaltered behaviour/movement, but habituated rapidly to the stimuli. In contrast, 40 kHz noise pulses consistently evoked rapid muscle flinches (indicative of startles), but no behavioural or heart rate changes. We conclude that the autonomous startle response appears decoupled from, or overridden by, cardiac regulation in diving porpoises, whereas certain novel stimuli may motivate oxygen-conserving cardiovascular measures. Such responses to sound exposure may contribute to gas mismanagement for deeper-diving cetaceans.

## Abstract (precis)

**Summary:** Trained porpoises respond initially to sonar sound exposure with intensified bradycardia, but habituate rapidly, whereas 40 kHz pulses consistently evoke a startle, but elicit no other behavioural or heart rate changes.

## INTRODUCTION

Naval sonar use has been linked to mass-strandings of beaked whales (e.g. Frantzis, 1998) and harbour porpoises (*Phocoena phocoena*) (Wright et al., 2013). While the mechanisms behind these strandings are still unknown, gas and fat emboli, indicative of decompression sickness (DCS), have been documented in stranded or drowned marine mammals including beaked whales and harbour porpoises (Jepson et al., 2003; Jepson et al., 2005; Moore et al., 2009; de Quiros et al., 2012; Siebert et al., 2013). While diving, increased peripheral vasoconstriction and proportionally decreased heart rate (*f*_H_) conserve blood oxygen, mainly for the brain and heart (Davis, 2019; Scholander, 1940). This dive response is influenced by dive duration and exercise (Davis and Williams, 2012; McDonald et al., 2018; Williams et al., 2015), and is also under anticipatory and volition control (Elmegaard et al., 2016; Elmegaard et al., 2019; Elsner et al., 1966). The dive response is thus highly dynamic to accommodate the instantaneous needs of the diving animal, and varies from extreme heart rate depression while escaping capture (Williams et al., 2017) to sometimes more subtle changes when swimming by the surface (Elmegaard et al., 2019; Scholander, 1940). While the dive response is important for O_2_ management, it also impacts N_2_ management in lungs, blood and tissues. Therefore, if a stressor alters the normal cardiovascular response, the risk of DCS may increase (Fahlman et al., 2014; Hooker et al., 2012). For example, decreased peripheral perfusion during ascent may reduce the flux of N_2_ from supersaturated tissues, increasing the risk of gas emboli during the decompression. While beaked whales and delphinids often cease sound production, change heading, dive deeper, and swim vigorously to avoid/escape mid-frequency sonar exposure (DeRuiter et al., 2013; Henderson et al., 2014; Houser et al., 2013; Tyack et al., 2011), it is unknown if these anti-predator responses to acoustic stressors (Tyack, 2011) are accompanied by physiological responses (i.e. fight-or-flight or freeze response) as seen for example in narwhals escaping a capture situation (Williams et al., 2017).

Generally, physiological defence responses to stressors can be categorised as active (fight-or-flight) or passive (orienting and freeze) responses (Knight and Gutzwiller, 1995) as well as more complex aversive startle and cardiac defence responses with different cardiac response regimes (Vila et al., 2007). While the cardiovascular response to an acoustic stressor in wild cetaceans has not been measured, the few studies on captive cetaceans have provided conflicting results with some individuals decreasing and others increasing *f*_H_, even within the same species. Such differing *f*_H_ response types may relate to naivety, sound characteristics, and context (Lyamin et al., 2016; Miksis et al., 2001; Teilmann et al., 2006). Cetaceans with a strong anti-predator response may be more likely to startle when exposed to certain noises (Wright et al., 2007). Sudden loud sounds are known to trigger the acoustic startle reflex, a mechanism thought to protect against sudden blows or attacks through transient whole-body muscle flinches, while preparing for a fight-or-flight response (e.g. reviewed in Koch, 1999). The reflex often involves cardiovascular responses characterised by an immediate transient acceleration of *f*_H_ followed by a slower deceleration (Vila et al., 2007). The involvement of both parasympathetic and sympathetic components can result in either bradycardia or tachycardia, depending on development, genetics, habituation, and emotional state (Baudrie et al., 1997, 2001; Berg and Beebe-Center, 1941; Chalmers and Hoffman, 1973; Globisch et al., 1999; Richardson et al., 1996; Svensson et al., 1991). The whole-body muscle flinches of the startle response are detectable by eye or characteristic jerks in accelerometer data, which are therefore often used as a measure of startle reflex activation and amplitude (Koch, 1999). Recently, whole-body muscle flinches in response to a startle sound have been documented in seals and cetaceans (Götz et al., 2020; Götz and Janik, 2011; Kastelein et al., 2012); however, *f*_H_ was not measured.

Here, we investigate the *f*_H_ and motor-response of porpoises exposed to both mid-frequency sonar-like sweep and startling noise pulse to understand the implications for a diving animal. Specifically, we test the opposing hypotheses that (1a) harbour porpoises exposed to mid-frequency sonar-like sweep playback respond with muscle flinches in concert with increased *f*_H_ for increased performance, or alternatively, (1b) respond with a cardiac freeze allowing prolonged breath-holding. To verify if a response is part of an autonomous startle-response, we hypothesised in a second set of experiments that (2) the response to a specially designed startle sound is characterised by muscle-flinches, and is accompanied by a transient *f*_H_ increase as seen in many terrestrial mammals.

## RESULTS AND DISCUSSION

### Sonar exposure

**Fig. 1.**
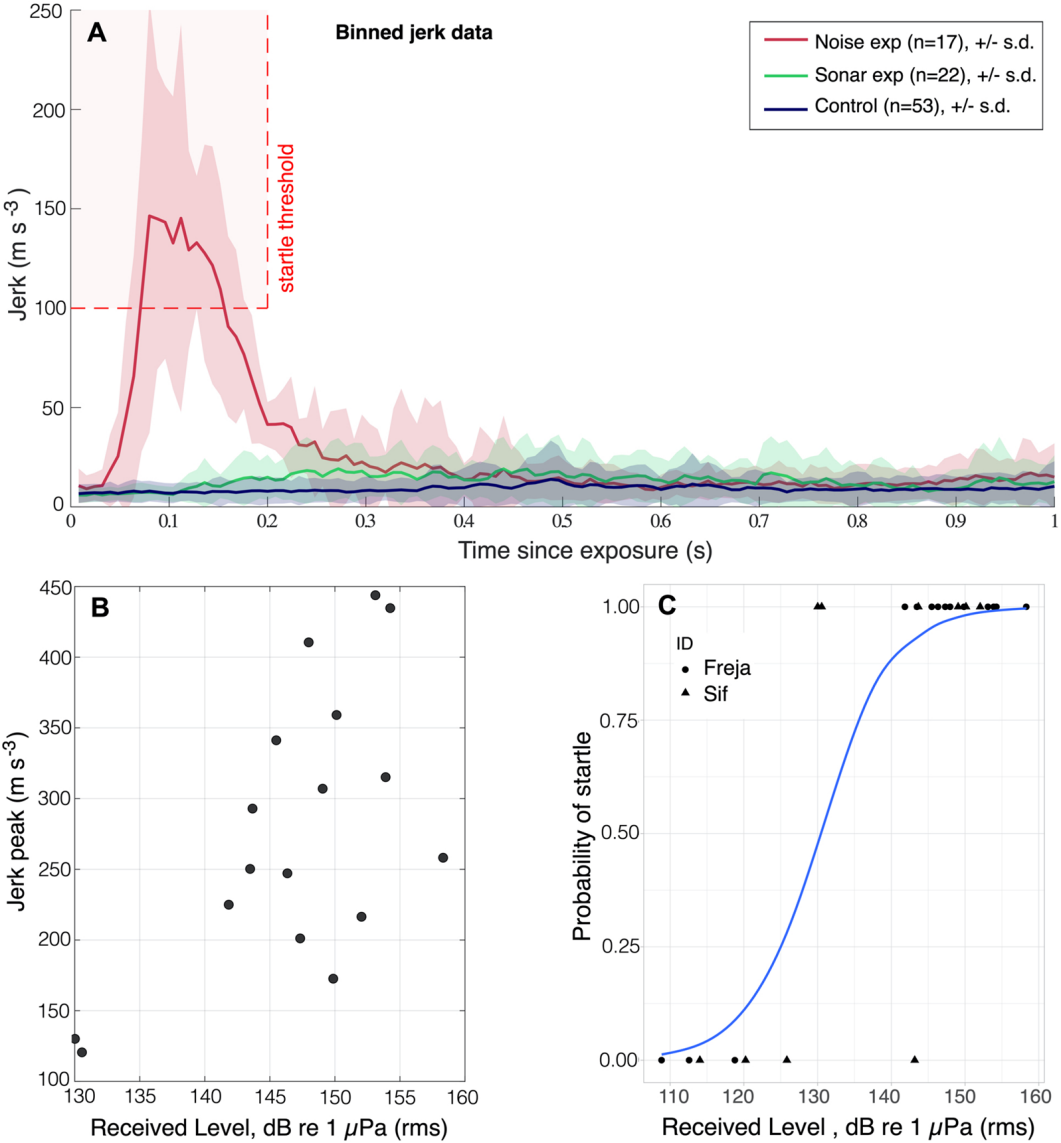
**Motor-responses to sonar and noise pulse exposures.** (A) Jerk data in 1/125 s bins with mean and standard deviation (s.d.) displayed for noise pulse exposure (red), sonar exposure (green) and controls from experiments together (blue). The threshold for startle detections (100 m s^−3^ within 0.2 s of exposure) is marked with red broken lines. (B) The startle jerk peak amplitude tended to increase with increased RL of noise pulses (rms_50_). Only exposures that resulted in a motor-response are included in plot. (C) Logistic regression analysis of startle probability with RL, including all noise pulse exposures, show a 50% startle chance at 130 dB re 1 µPa (rms_50_) for the 40 kHz noise pulse.

To investigate cardiac and behavioural response to sonar-like sweeps, the two porpoises completed 22 exposure trials (Freja *n*=13; Sif *n*=9) and 24 control trials (Freja *n*=15; Sif *n*=9). Received levels (RL) of the exposures [sound exposure level (SEL): 98-131 dB re 1 µPa^2^s, or rms_125_: 103-137 dB re 1 µPa] were ∼50–80 dB above the ∼55 dB re 1 µPa (rms) porpoise hearing threshold at 6–9 kHz (Kastelein et al., 2010) (Table 1). Therefore, we predicted a behavioural response as seen for a range of other anthropogenic noise sources at similar or lower loudness (i.e. RL relative to hearing threshold, Tougaard et al., 2015). However, we observed minimal or no difference in behaviour between control and exposure trials. After the first exposure, Sif mildly avoided initiating the task a few times, but once committed, she always ate the fish and returned to the trainer. Additionally, we did not detect any startle jerks, indicating that the sonar-like sweeps did not trigger the startle reflex (Fig. 1A; see also Fig. S3A). This could be due to the 50–100 msec rise time of the sonar-like sweep, which does not evoke an acoustic startle reflex in other mammals either (Blumenthal and Berg, 1986; Götz et al., 2020; Götz and Janik, 2011). Thus, there was no jerk response to the sonar-like sweep exposure (*P*=0.4, two-sample *t*-test comparing sonar and control; see Fig. S4C), but very low amplitude jerks were present in both exposure and control trials (one sample *t*-tests; sonar: 28.7% ±38.4, *P*=0.002; control: 20% ±35.4, *P*=0.01). They coincided with buzzes, not noise exposure, and were thus interpreted as prey capture jerks similar to, but at much lower amplitude than shown for live fish in Wisniewska et al. (2016).

**Table 1.**
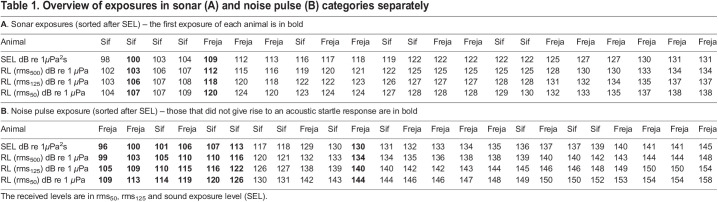
Overview of exposures in sonar (A) and noise pulse (B) categories separately

**Fig. 2.**
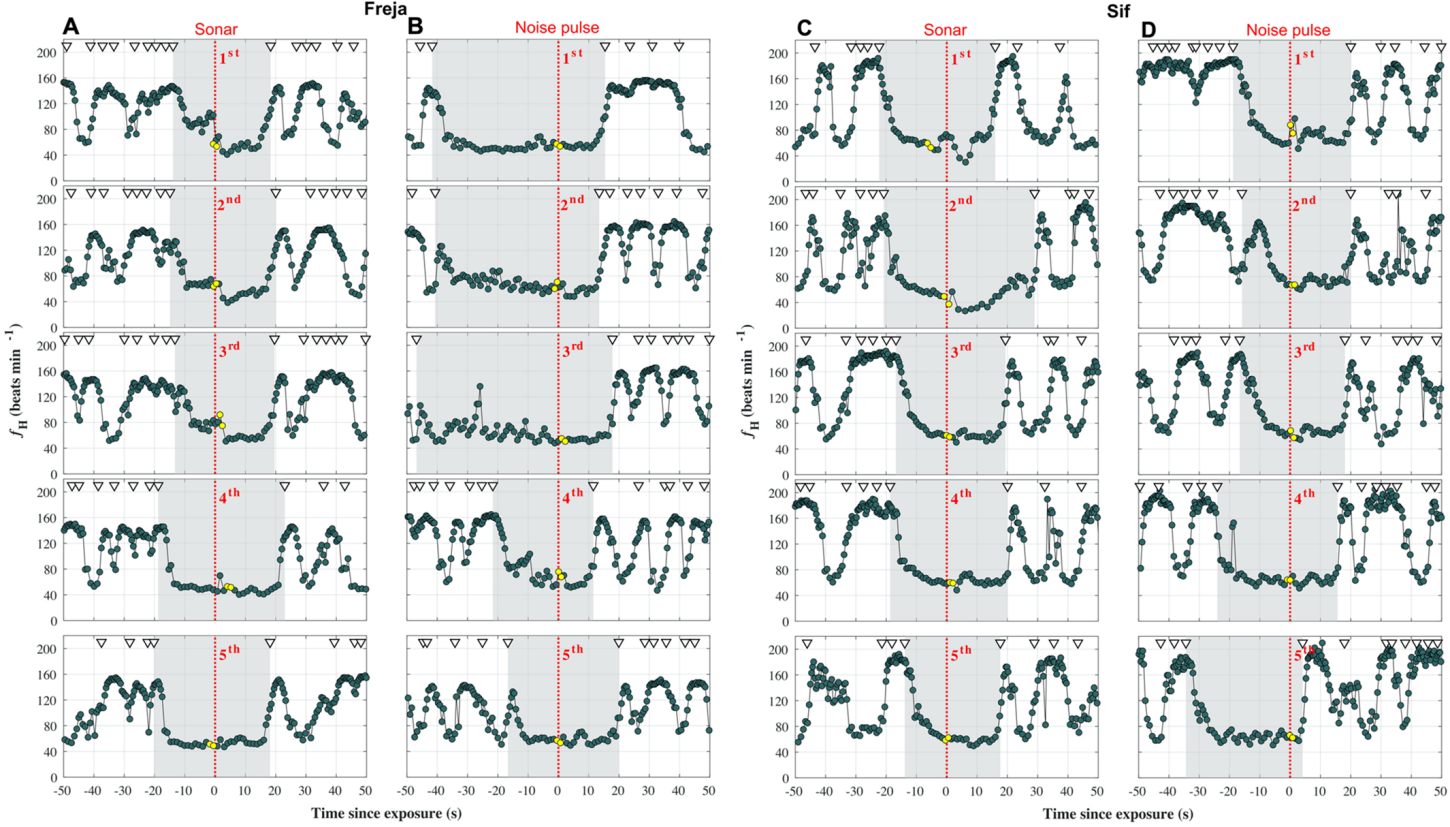
**Heart rate during initial exposures.** Instantaneous *f*_H_ traces during five first (A+C) sonar and (B+D) noise pulse exposures for Freja (A+B) and Sif (C+D), respectively. Grey areas mark trial dives, with onset from last breath before, and end at first breath when the porpoise was back with the trainer. By the end of dives, anticipatory tachycardia is evident just prior to breathing, as is typically observed in marine mammals (e.g. Hill et al., 1987; McDonald and Ponganis, 2014; Williams et al., 1999). Vertical lines mark the time point of sonar or noise pulse playback. Yellow coloured heart beats mark the buzz phase of the dead fish catch. Triangles in the top mark breaths. (A) At the first sonar exposure, Freja experiences a deeper bradycardia transiently (−61 beats min^−1^). From the fourth trial and onwards, no cardiac response was detectable. (B) In the noise pulse exposures, no cardiac responses were visible in spite of successful startle elicitations. (C) In Sif's first sonar-like sweep exposure, her *f*_H_ drops transiently (−43 beats min^−1^). In the second exposure, the decrease in *f*_H_ was smaller, and from the third exposure, a response was not detected. (D) In Sif's first noise pulse exposure, a transient increase in *f*_H_ was observed; however, such increase was not seen in any of the following noise pulse exposures.

**Fig. 3.**
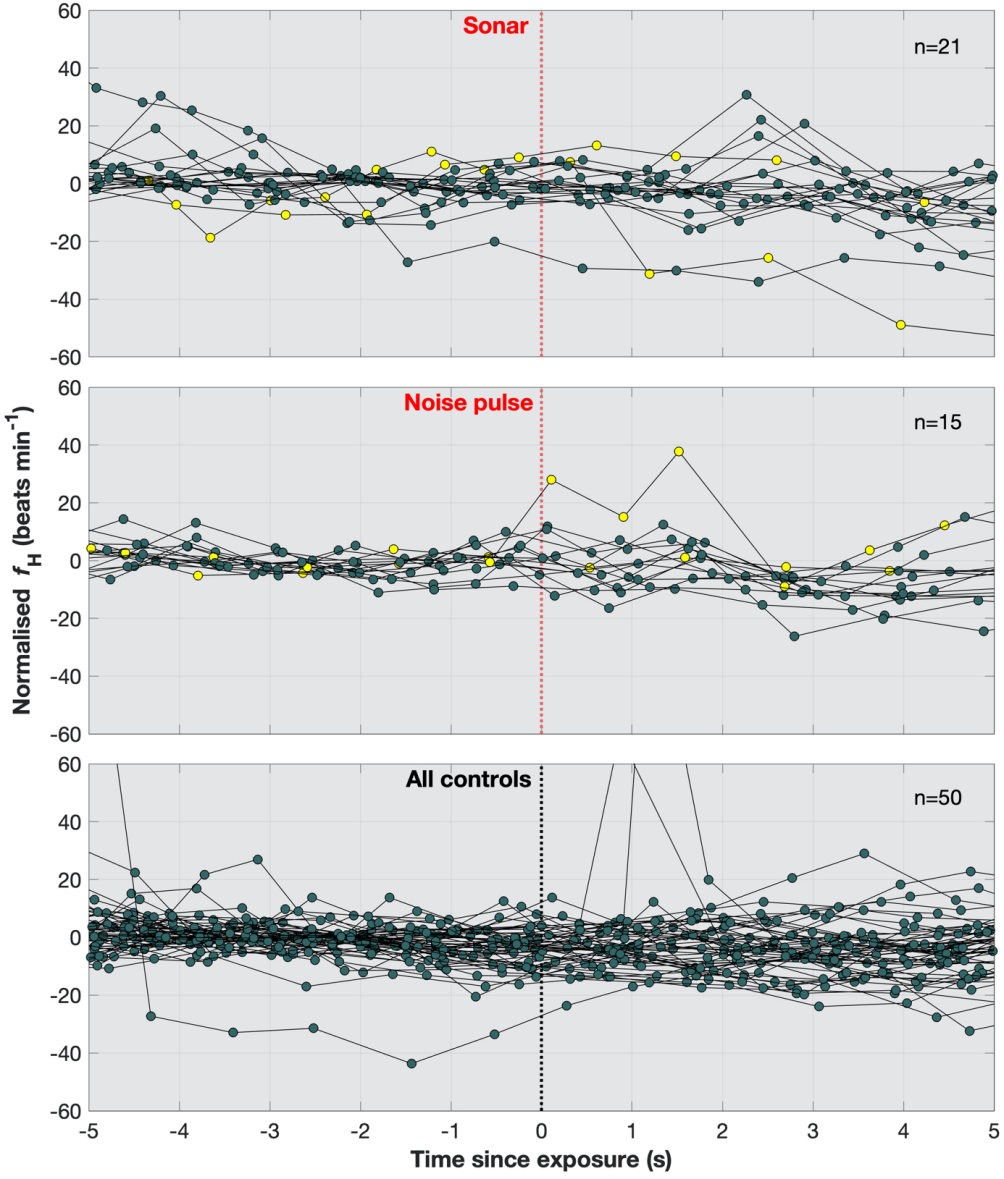
**Normalised *f*_H_ of all trials.** Traces normalised to 5 s mean preceding exposures, including data from both Freja and Sif from (A) sonar exposures, (B) noise pulse exposures that evoked startle responses, and (C) all control trials. The heart beats from the first exposure of each animal are coloured yellow in A and B. In sonar exposures, some *f*_H_ traces decreased at exposure, but habituation was fast and the overall trend was no change. In noise pulse exposures, *f*_H_ responses were not detected, in spite of motor response elicitation. Double beats, seen in two control trials as one-beat extreme peaks, are relatively normal in marine mammals.

We then tested the hypotheses that sonar exposure elicits either an acceleration or deceleration of *f*_H_, respectively, to either increase performance in a fight or flight response or prolong breath-hold potential. Even without a behavioural or movement response, the first sonar-like sweep exposure of each porpoise [RL, Freja: 109 dB re 1 µPa^2^s (SEL); and Sif: 100 dB re 1 µPa^2^s (SEL)] gave rise to transient 59–60% decreases of instantaneous *f*_H_ from last beat before exposure to minimum *f*_H_ within the following seconds. For Freja, the *f*_H_ decreased by 61 beats min^−1^ (Fig. 2A) and for Sif, 43 beats min^−1^ (Fig. 2C). Such a decrease could be indicative of an orienting response to a new stimulus (Vila et al., 2007). For Freja, the drop was from an elevated *f*_H_ at the time of exposure, compared to later trials. For Sif, the starting *f*_H_ at exposure time was similar to other trials, so the *f*_H_ dropped below average diving *f*_H_. Although the first two to three exposures initiated a clear decrease in *f*_H_, the responses diminished with succeeding trials (Figs 2A,C and 3A), and resulted in a small, but significant change in *f*_H_ from mean 5 s pre-exposure to mean 5 s post-exposure (−6.6 beats min^−1^ ±11.3, *P*=0.015, one-sample *t*-test). However, there was no difference in the magnitude of the *f*_H_ decrease between sonar and control trials (*P*=0.053, Welch's two-sample *t*-test; see Fig. S4A, and control trials in Figs S1A and S2A). Thus, the porpoises habituated quickly to the sonar-like sweep exposures. Even after a 3-year pause between sonar exposure sessions, Freja did not decrease *f*_H_ like in her first few sonar-like sweep exposures. In 1999, Freja's *f*_H_ and behaviour was documented in response to pinger-like sounds (100–140 kHz) (Teilmann et al., 2006). The first exposure then intensified her diving bradycardia, but there was no *f*_H_ response in following exposures despite avoidance of the sound source. Furthermore, in restrained captive belugas exposed to noise playbacks of a variety of frequencies, the first exposure had clear effects, albeit different, in three individuals: One responded with bradycardia, one with tachycardia, and one with a narrower range of *f*_H_. After repeated exposures, the cardiac response lessened for all three individuals (Lyamin et al., 2016). While habituation of heart rate responses occurred rapidly in captivity, it is unknown if, or at what pace, this will occur in the wild, where acoustic stimuli often are novel and less predictable and where animals can flee. Drastic and sustained *f*_H_ response to novel sounds may be common in species with high predation rates or that tend to be shyer in accordance with the risk disturbance hypothesis: Such species are more likely to perceive novel sounds as threats (Tyack, 2011). The captive porpoises have participated in a variety of acoustic and behavioural studies, and have developed trust with the trainers; therefore, they may likely display lower responsiveness and faster habituation than wild naïve cetaceans. Behavioural habituation to continuous and periodical pinger exposure has been demonstrated in several passive acoustic monitoring studies of wild harbour porpoises (Cox et al., 2001; Kindt-Larsen et al., 2019; Kyhn et al., 2015), suggesting that the wild porpoises may display some resilience to continued or repeated sound exposure. It is important, however, to distinguish between behavioural and physiological responses, and habituation and tolerance: Stressor presence may have a cost for the animals in spite of continued use of habitat (Bejder et al., 2009). This is supported by the observed initial heart rate responses to sonar-like sweep exposures without any behavioural change (Fig. 2A,C). While we see rapid habituation within a few exposures, it remains to be seen if physiological response habituation also occurs in the wild.

The initial bradycardia response to sonar-like sweeps allows a prolonged breath-hold to assess the nature of a novel stimuli or flee in crypsis if needed. The intensified dive response is consistent with experiments on a harbour seal (*Phoca vitulina*) exposed to a variety of sudden novel stimuli: There, peripheral blood pressure measurements revealed that it responded equally to all stimuli by limiting peripheral blood flow thus conserving blood oxygen for potentially prolonged dive times (Irving et al., 1942). This supports the idea that a general response in marine mammals to novel stimuli and potential threats may be a conservative orienting approach with intensified bradycardia and limited peripheral blood flow, as also documented in escaping narwhals with cardiac freezes (Williams et al., 2017). Based on the observed response to sonar-like sweeps at relatively low RLs, it is possible that naïve wild cetaceans will have a more pronounced and extended cardiovascular response when exposed to powerful naval sonar, even at long ranges. If the response is an intensified bradycardia (and lower peripheral perfusion), the N_2_ diffusion from the tissues to the blood and lungs will be diminished during ascent, potentially putting the animal at higher risk of DCS if tissues are supersaturated (Fahlman et al., 2014). Less acute effects of gas mismanagement may increase recovery time at the surface, resulting in less time available for foraging per day. This could in extreme cases compromise the individual's energy budgets.

### Noise pulse exposure

**Fig. 4.**
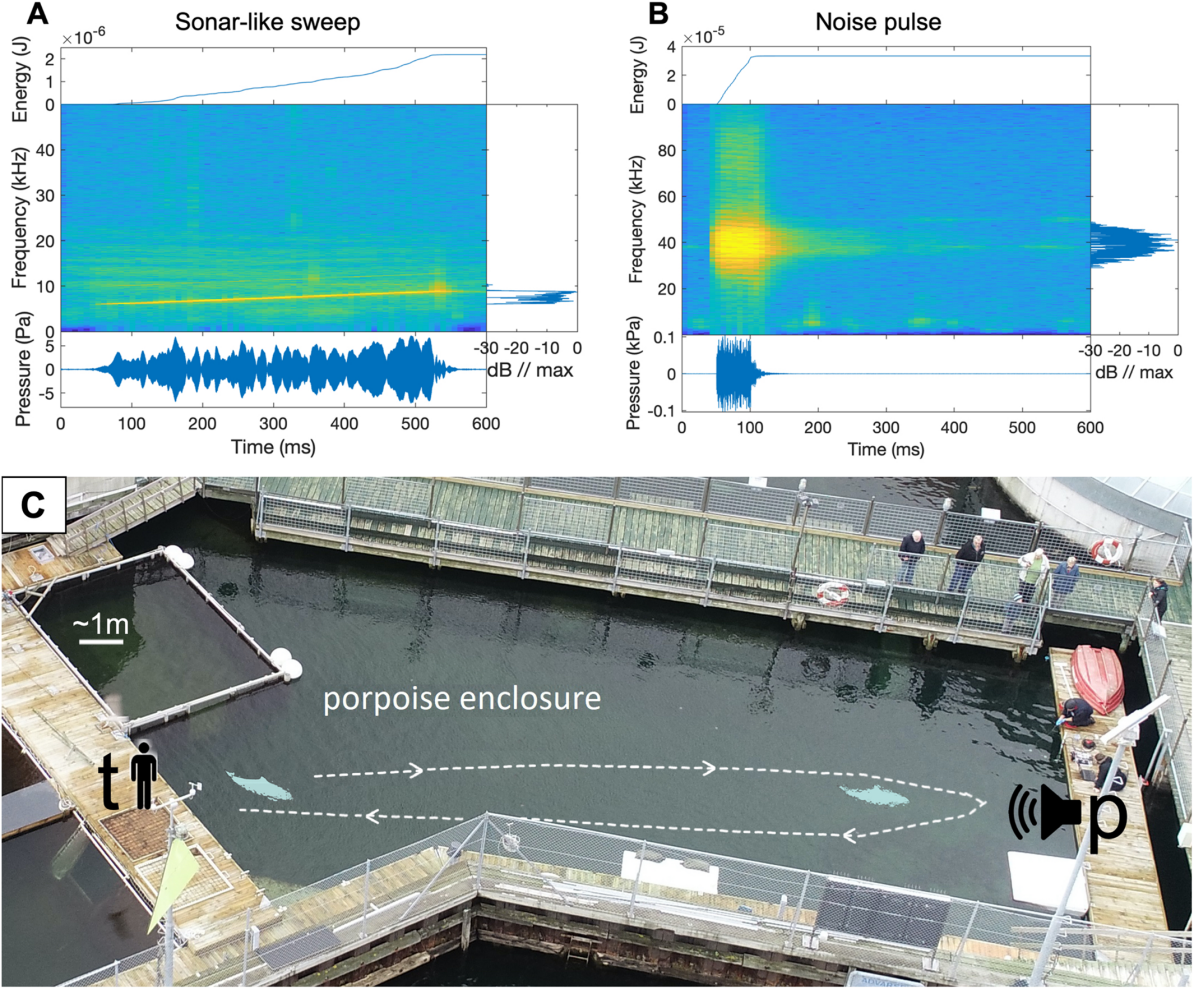
(**A)** **Sonar-like sweep and (B) noise pulse characteristics after running a broad band-pass filter (4–90 kHz) on sounds recorded on tag.** The 6–9 kHz sonar-like sweep has a duration of 500 msec, while the noise pulse is centred at 40 kHz with a duration of 50 msec. Accumulated energy plots (top subplot in both A and B) show that the noise pulse exemplified has about 15 times more energy than the sonar-like sweep exemplified (notice the values of the axes). The main frequency components are depicted in frequency spectrum plots to the right of A and B. The waveforms are in the bottom sound pressure level plots. The spectrograms in the centres sum up both the frequency content with colour coded intensities, and timeframe of the sound recordings. (C) Overview of the Fjord & Belt porpoise pen, where the study was conducted. The trainer position (t) and playback station position (p) are marked. A ∼1-m scale bar is shown in top left corner. Photo courtesy of M. Wahlberg. Edited by S. Elmegaard.

We exposed porpoises to a broadband noise pulse (Fig. 4B) to investigate if the porpoises exhibit the typical startle motor-response and associated increase in *f*_H_ in preparation for fight or flight [exposures: *n*(Freja)=15, *n*(Sif)=9; controls: *n*(Freja)=19, *n*(Sif)=10]. As observed in sonar-like exposures, neither porpoise displayed aversive behaviour in the trials. Freja hesitated to initiate one trial, but no trials were aborted once committed. Seven exposures did not evoke a startle response [RL: 109-143 dB re 1 µPa (rms_50_), or 96–130 dB re 1 µPa^2^s (SEL)], whereas 17 did [*n*(Sif)=6; *n*(Freja)=11; RL: 130–158 dB re 1 µPa (rms_50_), or 117–145 dB re 1 µPa^2^s (SEL)] (Fig. 1A; see also Fig. S3B). In trials with a startle motor-response, mean jerk after exposure was elevated by 43% ±52 (*P*=0.0035, one-sample *t*-test; see Fig. S4D). In the control trials, there was no difference in mean jerk before and after exposure (−5.6% ±32, *P*=0.36, one-sample *t*-test). This resulted in a significant effect of exposure compared to control (*P*=0.002, Welch's *t*-test for unequal variance). The startle motor-response amplitude was positively correlated with RL (Fig. 1B), and higher RL were more likely to evoke a response (Fig. 1C), in line with other studies (e.g. Blumenthal and Berg, 1986; Götz et al., 2020; Götz and Janik, 2010). The startle response was evoked at ∼85-113 dB above hearing threshold (∼45 dB re 1 µPa rms for 40 kHz tones, Kastelein et al., 2010), with a 50% motor-startle probability threshold around 130 dB re 1 µPa (rms_50_). This response threshold (∼85 dB above hearing threshold) is similar to the response threshold observed in bottlenose dolphins (∼90 dB over hearing threshold) (Götz et al., 2020). The time to muscle flinch onset is shorter in the porpoises (<0.1 s) than in the dolphins (0.2–0.3 s), reflecting the smaller size and shorter transmission distance in the reflex arcs of the porpoises.

In spite of a clear motor response, the noise pulse exposures did not evoke general *f*_H_ changes in the porpoises (−3.4 beats min^−1^ ±5.5, *P*=0.25, one-sample *t*-test; see Fig. S4B), resulting in no difference between exposure and control trials (*P*=0.44, two-sample *t*-test) (Figs 2B and 3B; see control trials in Figs S1B and S2B). In Sif's first noise pulse exposure, she exhibited a transient *f*_H_ increase (Fig. 2D). From terrestrial animals, cardiac responses of startles are known to habituate to some degree if the stimulus is replayed in close proximity, but is also known to be repeatable with a recovery time between stimuli similar to the one we employed with the porpoises (Chalmers and Hoffman, 1973; Vila et al., 2007). Thus, since the *f*_H_ increase was only observed in the first exposure, it is evidently not an obligatory or dominant component of the acoustic startle reflex in porpoises. Even though startles manifest with transient cardiac acceleration in many terrestrial mammals (Chalmers and Hoffman, 1973; Vila et al., 2007), while diving, there is a strong parasympathetic tone to the heart (Ponganis et al., 2017), which could simply override transient sympathetic stimulation, or in some cases may not leave much parasympathetic regulatory room for further cardiac depression. In the porpoises, however, the vagal tone is probably not maximal, since much lower *f*_H_, below 15 beats min^−1^, have been recorded from these animals previously (McDonald et al., 2018). Therefore, if anything, the startle could induce a transient, but overridden, increase in sympathetic or release of parasympathetic tone to the heart, where Sif's initial exposure perhaps induced a stronger response than in the following exposures; or, diving mammals have eliminated a cardiac startle response, allowing for stable volitional cardiovascular regulation while breath-holding at depth.

In conclusion, the two captive harbour porpoises displayed intensified bradycardia at initial sonar-like sweep exposures. The following trials did not evoke the same *f*_H_ response, even after a 3-year pause, suggesting a very rapid and lasting habituation. The exposure received levels did not cause simultaneous behavioural responses, as predicted from wild porpoise exposure data (Tougaard et al., 2015). Acoustic startle motor responses were successfully evoked by noise pulse exposures, but there was no associated *f*_H_ increase as often seen in terrestrial mammals (Koch, 1999). Thus, it seems that the dive response of the harbour porpoises overrides potential sympathetic startle waves, or that they have evolved to decouple cardiovascular changes from autonomous startle reflexes. This may be essential for proper O_2_ and N_2_ management. Yet, as seen from sonar-like sweep exposures, porpoises may still display cardiac responses to novel stimuli, the details of which is probably dependent on individual experience, context and perceived threat. While the observed decrease in *f*_H_ was transient and subtle, more prolonged or repeated responses would give them more time at depth to assess and escape, while paradoxically potentially putting them at increased risk of DCS if occurring upon ascent with supersaturated tissues. We advocate that more studies should be performed at sea with heart-rate logging multi-sensor tags to elucidate if or how wild cetaceans may implement the documented physiological responses to actual exposure under conditions of high ecological validity, while often foraging and navigating a landscape of fear.

## MATERIALS AND METHODS

### Data collection

Data were collected from two trained harbour porpoises, at the Fjord and Belt Centre in Kerteminde, Denmark, during November to December 2014 (Freja and Sif), and repeated with one of these porpoises in December 2017 (Freja). The porpoises were housed in a 15×35 m net-pen under permits from the Danish Council for Experiments on Animals and the Ministry of Environment and Food of Denmark (SN 343/FY-0014 and 1996-3446-0021) and with experimental approval from the IACUC of Aarhus University.

The animals were trained to wear multi-channel dataloggers (ecg-DTAG3, see McDonald et al., 2018) that were attached with suction cups to the back of the porpoises ∼5 cm behind the blowhole. Two versions were used during the study. For both versions, two electrodes embedded in suction cups were attached to the porpoise. The electrode on the right side was placed rostral to the heart and the left electrode was placed caudal to the heart. The datalogger recorded the differential potential between the two electrodes relative to a ground in water, creating the electrocardiogram [ECG - sampling rate: 10 kHz (tag1) or 5 kHz (tag2), 16-bit resolution and 2-pole 200 Hz anti-alias filter], along with measurement of three-dimensional (3D)-acceleration [sampling rate: 2 kHz (tag1) or 625 Hz (tag2)], and stereo sound (500 kHz sampling, 16-bit resolution, 0.5-150 kHz bandwidth). This gave synchronised measures of *f*_H_, 3D movements, and RL of sound exposures.

We created a 500 msec 6–9 kHz tonal sweep (Fig. 4A) with a measured rise time of ∼50–100 msec to simulate the sound of a mid-frequency naval sonar linked to cetacean strandings (e.g. Frantzis, 1998). In an attempt to induce a startle response, we also created a 50 msec noise pulse (peak frequency 40 kHz, half power bandwidth of ∼5 kHz) with a rapid rise time (<5 msec). Such a pulse has characteristics similar to many echosounder pulses, and to pulses that have been broadly applied in the literature to induce the acoustic startle response in a range of animals (e.g. Chalmers and Hoffman, 1973; Götz et al., 2020) (Fig. 4B). The porpoises were exposed to the noise pulse at levels that were ∼65–115 dB above hearing threshold at 40 kHz for harbour porpoises (∼45 dB re 1 µPa, Kastelein et al., 2010). We designed the noise pulse based on studies of seals and rodents, where sound rise times were negatively correlated, and bandwidth positively correlated to startle muscle flinch magnitude and probability (Blumenthal and Berg, 1986; Götz and Janik, 2011). In studies of seals and dolphins, a startle response was induced at 80–90 dB above hearing threshold (Götz et al., 2020; Götz and Janik, 2011). During the first trials, the sounds were played at lower source levels in a conservative approach, and then ramped up with progressing trials. This maximised response likelihood while we monitored the behaviour of the animals to ensure that they were not unintentionally affected by the exposure.

For each session, a trainer was located at one end of the pool, and a playback station with underwater speakers at 1 m depth was located at the other end of the pool, approximately 35 m from the trainer (Fig. 4C). The playback was controlled from a custom-made program (using LabView, National Instruments, Austin, TX, USA) running on a laptop. The laptop was connected to speakers through a NI-box (National Instruments, Austin, TX, USA) with two outputs. For the sonar playback one output was connected to a Rockwood AM-2120 120-Watt amplifier (Rockwood, USA) and a Lubell EV UW30 underwater-speaker (Lubell Labs Inc., Columbus, OH, USA). For the noise pulse (startling sound) playback, which was higher frequency and played at higher sound pressure levels (peak-peak, to achieve similar sound energy levels), the second NI-box output was connected to a custom power amplifier and spherical hydrophone (Sonar products HS26, Driffield, UK). Calibrated SoundTraps (Oceans Instruments, New Zealand) were placed 1 m from the speaker to monitor output level. Additionally, we played back empty sound files to control for artefact from the experimental setup.

For each trial during a session, the trainer sent the porpoise to the speaker-end of the pool to eat a dead fish that was thrown approximately 1 m in front of the speaker by a second person. The porpoise then returned to the trainer for further rewards. During this activity the porpoise swam at ∼1 m depth while breath-holding for 30–45 s. For some trials the porpoise was fed several fish before the exposure, resulting in longer breath-holds. The sonar, noise pulse, or control was played when the porpoise was approximately 1–2 m in front of the speaker, just before or as the porpoise reached the fish. Between trials, the porpoise had at least 1 min in minimal activity at the surface for full metabolic recovery between dives. Sessions were run with one porpoise at a time and with sonar-like sweeps or noise pulse exposures in separate sessions. Each session consisted of ∼50% control and ∼50% exposure trials in random order.

### Data processing

Data were processed using custom-written scripts in MatLab (The MathWorks, Natick, MA, USA). ECG data were down-sampled and bandpass-filtered for better automatic detection of R-peaks in the QRS-complex, which were then visually checked. Instantaneous *f*_H_ per beat was calculated from the time difference between an R-peak and the previous. Buzzes (the final echolocation phase before catching the fish) were manually marked by visual inspection of spectrograms (Hamming window, fast Fourier Transform size 512, 75% overlap). To assess for startle-twitches in the form of rapid movements associated with sound exposures, we calculated norm-jerk, i.e. the square root of the summed squared triaxial differential accelerations (Ydesen et al., 2014).

Received sonar-like sweeps and noise pulses, recorded on-animal, were band-pass-filtered (4–90 kHz) to remove low frequency noise as well as the majority of echolocation energy (110–150 kHz, Møhl and Andersen, 1973). For sonar-like sweeps, the 125-msec window with maximal energy was used to calculate the root mean square value of sound pressure level (rms_125_ or Leq-fast, dB re 1 µPa) to compare with hearing thresholds from Kastelein et al. (2010). To accommodate the short duration of the noise pulse, a 50-msec window was used for these rms calculations (rms_50_, dB re 1 µPa). Furthermore, to compare energy levels between sonar-like sweeps and noise pulses, sound exposure levels (SEL, dB re 1 µPa^2^s) were calculated by integrating the rms intensities with the durations of the sounds (Madsen, 2005). See Table 1 for all calculated values. None of the received levels were close to levels that can induce temporary threshold shifts in porpoises (Lucke et al., 2009).

### Data analysis

To determine if porpoises exhibited a startle motor-reflex in response to the broadband noise pulse we examined the first 0.5 s of the jerk data following the acoustic stimuli. Using half of the data, we defined a startle threshold as a jerk exceeding 100 m s^−3^ within 0.2 s of the onset of sound exposure. This threshold definition was then applied to the second half of the broadband pulse trials, resulting in satisfactory detections. To determine the risk of false positives with the startle threshold detector, the startle detector was run on the 29 control-trials. Four startle reactions were detected in three trials when looking at a 10 s window of jerk data. This gives a false positive detection of 0.003 startles per 0.2 s interval [4/(29×10×5)=0.003], or 0.05 probability of detecting a false positive in the 17 positive startle trials (17×0.003=0.05), which is highly unlikely.

To examine the relationship between stimulus level and probability of a motor-response to the noise pulse, we used a logistic regression model in R (R v.3.6.2, R Foundation for Statistical Computing, http://www.R-project.org/), i.e. a GLM with binomial error distribution. The independent variable was received level and the response variable was whether the porpoise exhibited a motor-startle response (binomial: yes/no). A GAM-curve was fitted to display startle probability related to RL, and allowed reading of a 50% response value.

To compare exposure and control trials, we needed to determine the specific time points of comparison. While the timing of exposure was easily determined from the sound recordings on the tag, the timing of the control playback could not be determined as such. Because the control trials needed to reflect approximately the same point of the stereotypical behaviour, we estimated exposure times relative to the event of buzzing (i.e. catching the fish near the playback location), which were normally distributed. The control time points relative to the buzz were then selected at random from a distribution with the same mean and variance.

To test the effect of exposure on *f*_H_ and jerk we calculated the relative change in jerk and absolute change in *f*_H_ from the 5 s pre-exposure mean to the 5 s post-exposure mean for each trial. We report mean±s.d. For noise pulse exposure trials, only positive startle responses were used (17 of 24 trials). Each group (e.g. sonar exposure jerk change, noise pulse control *f*_H_ change, etc.) was tested for normality, and equal variance between corresponding groups (e.g. sonar exposure *f*_H_ change and sonar control *f*_H_ change) was tested using a two-sample *F*-test. We tested the null-hypotheses that the groups were equal to zero (i.e. no change from pre- to post-exposure) using one-sample *t*-tests, and we tested the null-hypotheses that the exposure and control groups were from distributions with equal means (i.e. no difference between exposure and control treatments) using Welch's *t*-test for unequal variance or a two-sample *t*-test for equal variances (sonar jerk exposure and control). Trials were excluded from *f*_H_ analyses if the porpoise took a breath in the 10 s prior to or after exposure, since porpoises display a strong respiratory sinus arrhythmia that could influence *f*_H_ means. This resulted in the exclusion of one control and one exposure trial from the sonar *f*_H_ analysis, and two controls and two exposure trials from the noise pulse *f*_H_ analysis. For visualisation of exposure-induced changes, *f*_H_ data was normalised to the mean *f*_H_ of the 5 s preceding exposures or control-exposure times.

## Supplementary Material

Supplementary information
