# Supplementary material for: Heart rate and startle responses in diving, captive harbour porpoises (Phocoena phocoena) exposed to transient noise and sonar
Source: Biol Open. 2021 Jun 16;10(6):bio058679. doi: 10.1242/bio.058679 (PMC8249908; doi:10.1242/bio.058679)
Supplement: Supplementary information [file biolopen-10-058679-s1.pdf]

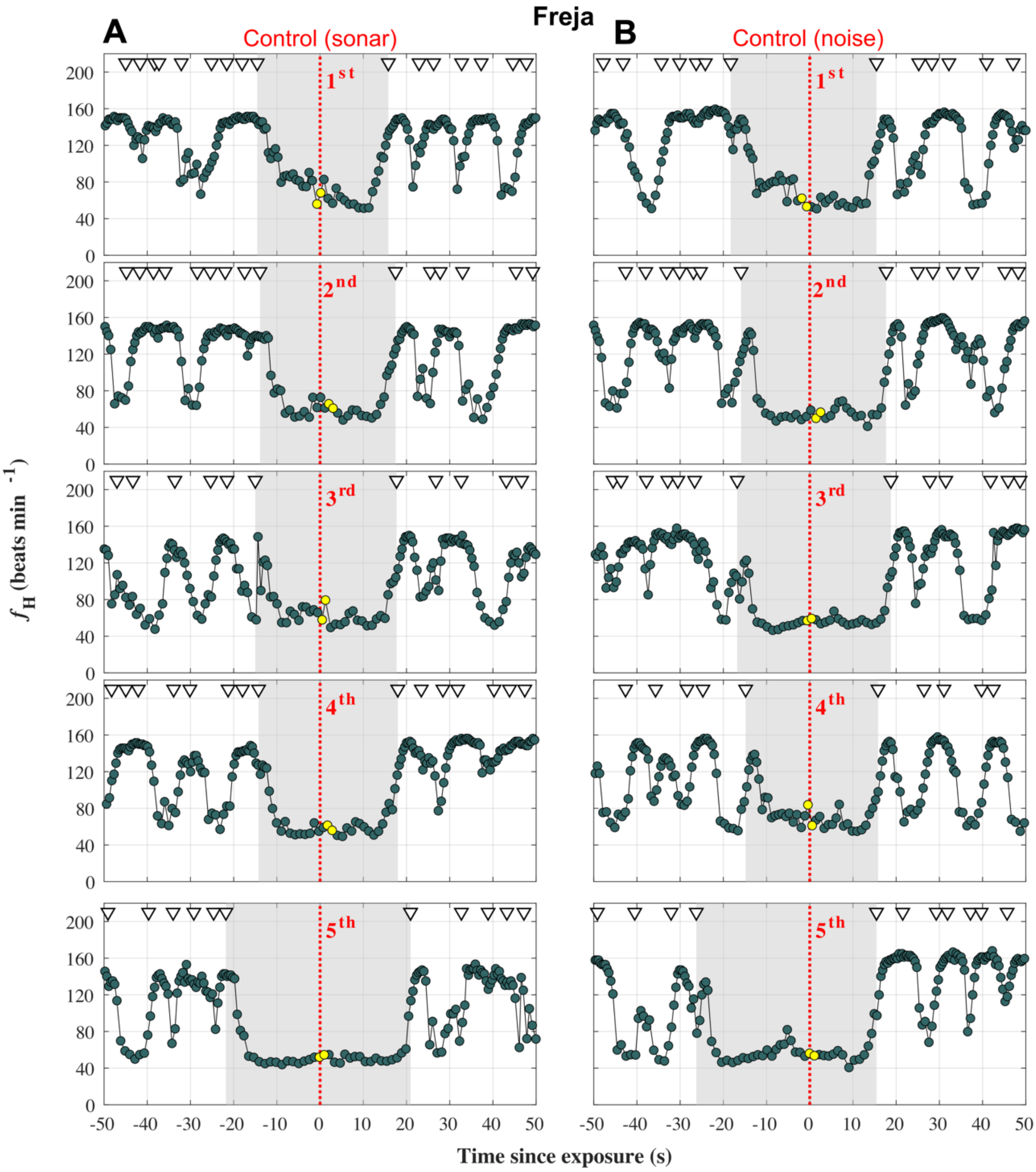

Fig. S1. Five first control trials for both Freja's sonar (**A**) and noise pulse (**B**) experiments.

Biology Open • Supplementary information

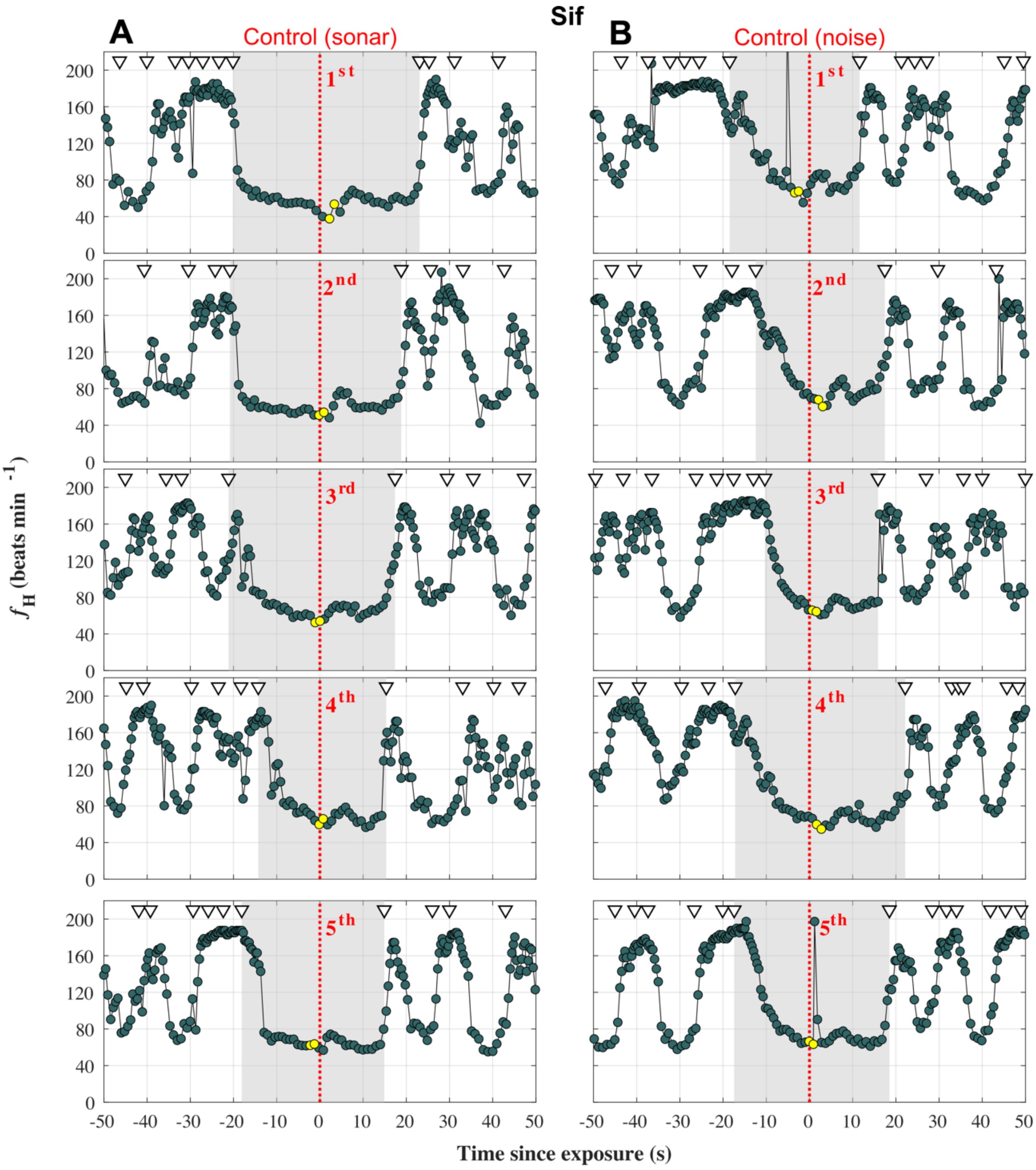

Fig. S2. Five first control trials for both Sif's sonar (**A**) and noise pulse (**B**) experiments.

Biology Open • Supplementary information

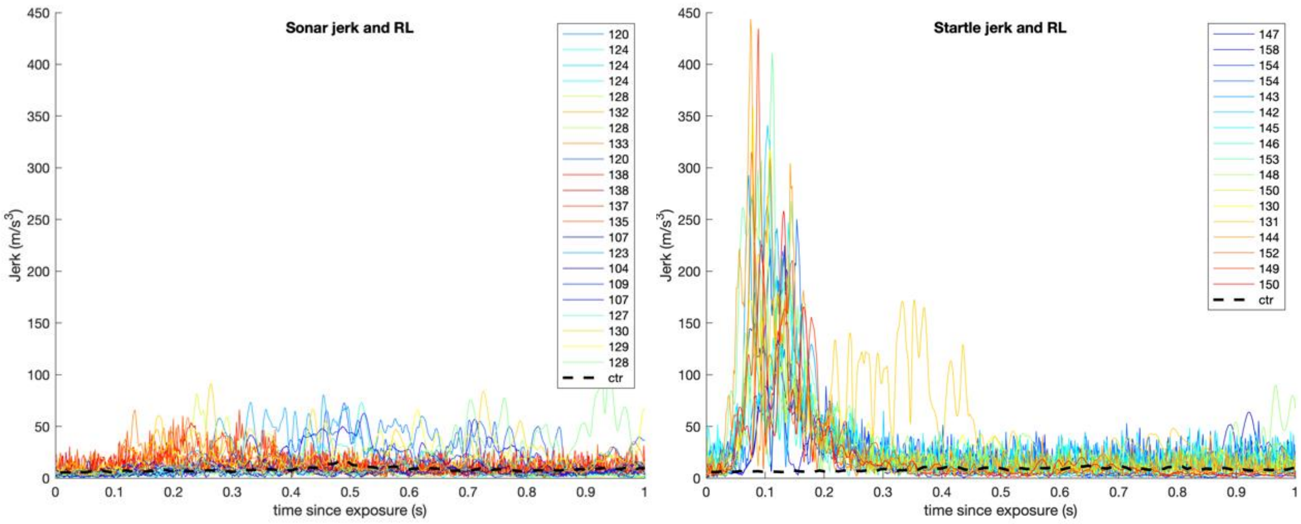

A **B**

Fig. S3. Raw jerk data for (**A**) sonar and (**B**) noise pulse exposure trials. Control data is displayed as a curve of the binned mean (broken line). Received level (rms_{50}) of each trial is displayed in legend box.

Biology Open • Supplementary information

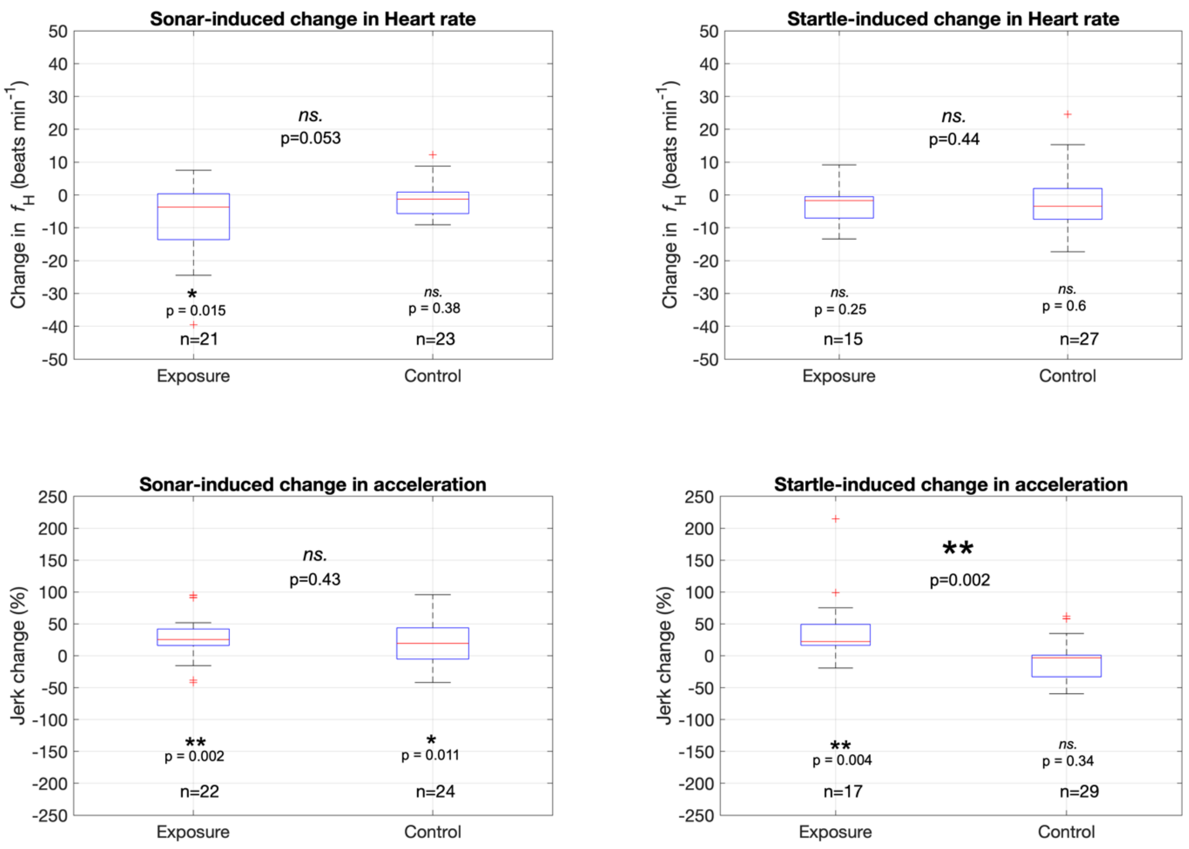

Fig. S4. Boxplot of data used in t-tests to test for effect of exposure. Below the boxes are the p-values for the one-sample t-test of the distribution compared to 0. Between boxes are p-values from the two-sample t-test (**C**) or Welch's t-test (**A, B, D**) for comparison of exposure and control data. Null-hypotheses were rejected at the 0.05 significance level. P-levels indicated as follows: ns. not significant, * p≤0.05, ** p≤0.01, *** p≤0.001.

Biology Open • Supplementary information
